# Association of type IV spinal muscular atrophy (SMA) with myoclonic epilepsy within a single family

**DOI:** 10.1186/1755-7682-7-42

**Published:** 2014-09-26

**Authors:** Damith S Liyanage, Lakmini S Pathberiya, Inuka K Gooneratne, Kumarangie K Vithanage, Ranjanie Gamage

**Affiliations:** Institute of Neurology, National Hospital of Sri Lanka, Colombo, Sri Lanka

**Keywords:** Spinal muscular atrophy, Myoclonic epilepsy

## Abstract

**Background:**

Spinal muscular atrophies (SMAs) are a group of disorders characterized by degeneration of the anterior horn cells in the spinal cord and motor nuclei in the lower brainstem. It is transmitted by autosomal recessive inheritance and most of these conditions are linked to SMN gene. Even if the clinical picture is mainly dominated by the diffuse muscular atrophy, some patients can also show atypical clinical features such as myoclonic epilepsy (“SMA plus”), which may be related to other genes. In particular, the association of SMA and progressive myoclonic epilepsy (PME) has been previously described.

**Case presentation:**

We present a case of two brothers with late onset SMA associated with a unique form of non progressive myoclonic epilepsy without cognitive impairment or ataxia. They had identical clinical and electrophysiological features.

**Conclusions:**

The association of SMA with myoclonic epilepsy may constitute a separate and genetically independent syndrome with unique clinical and electrophysiological findings. Collection of similar cases with genetic studies is needed to define the phenotype clearly and to identify new genes and molecular pathogenetic mechanisms involved in this condition.

## Background

Spinal muscular atrophies (SMAs) are a group of disorders characterized by degeneration of the anterior horn cells in the spinal cord and motor nuclei in the lower brainstem. It is transmitted by autosomal recessive inheritance [1]. They are classified as types I through IV depending on the age of onset and the clinical course. SMA type I, also known as infantile spinal muscular atrophy or Werdnig-Hoffmann disease, is the most common and severe type of SMA. It typically presents in the neonatal period. SMA type II (intermediate form) presents between 3 and 15 months of age, whereas SMA type III (Wohlfart–Kugelberg–Welander disease) typically presents with signs of weakness at or after 1 year of age and progresses to a chronic course. Adult onset of SMA (type IV) usually presents in the second or third decade of life and is otherwise similar to SMA type III [1]. Even if the clinical picture is mainly dominated by the diffuse muscular atrophy, some patients can also show atypical clinical features (e.g. oculomotor palsy, epilepsy, nerve deafness, olivopontocerebellar atrophy, multiple arthrogryposis, etc.) [2]. In particular, the association of SMA and progressive myoclonic epilepsy (PME) has been rarely described. In the last few years, experts particularly focused attention on the so-called “SMA plus” phenotypes and suggested clinical and genetic heterogeneity [3]. Here we describe a case of two brothers with type IV spinal muscular atrophy (SMA) associated with a unique form of non progressive myoclonic epilepsy, who had identical clinical and electrophysiological features. They never had ataxia or cognitive impairment to suggest a syndrome of Progressive Myoclonic Epilepsy (PME). PMEs are genetically heterogeneous group of disorders characterized by seizures, myoclonus, ataxia, and dementia, usually leading to severe mental impairment and disability.

## Case presentation

Two brothers belonging to ages of 28 and 22 years, presented with bilateral symmetrical lower and upper limbs wasting and weakness where the onset of the disease had been in early second decade of their life. They were born to healthy non consanguineous parents. Difficulty in combing hair, walking, running and getting up from squatting position were the prominent symptoms, which had been progressive. They were associated with arm and thigh muscle wasting. During the course of the disease they developed difficulty in fine movements such as buttoning, unbuttoning and writing. They never had deafness, diplopia, dysarthria or dysphagia. Both the patients developed myoclonic jerks involving bilateral upper limbs and head nodding without loss of consciousness, at the ages of 18 and 17 respectively. They were most prominent on action and disappeared during sleep. Frequency was 10 to 20 times a day and they were non progressive. There was no evidence of other seizure types. They had no history of cognitive decline or unsteadiness. Family history was positive for progressive muscular weakness and wasting in maternal uncle, who had died at the age of 20 due to a road traffic accident. He had no history of myoclonus and had not been investigated until his death.

Neurological examination showed diffuse muscle weakness and wasting, involving both upper and lower limbs and girdle muscles. They were more marked in proximal muscle groups. Elder of the two had scoliosis. Tendon reflexes were diminished in both of them. They had no facial weakness, ophthalmoplegia or hearing deficit. Fundoscopy was normal. There were no cerebellar signs. Cognitive functions of both of them were normal.

**Figure 1:**
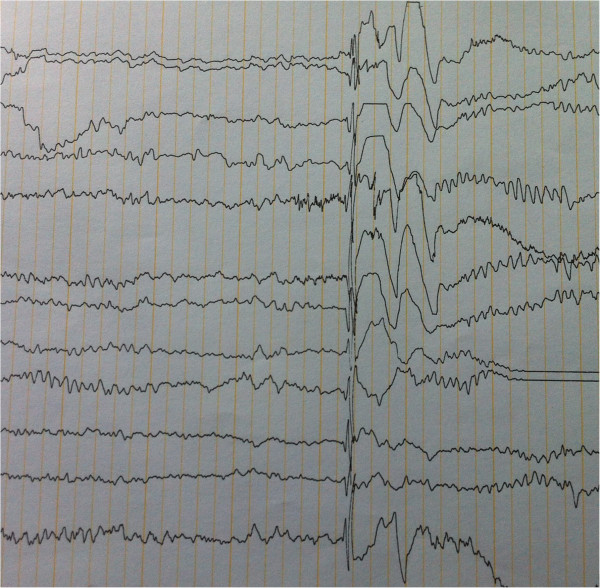
**EEG of the elder brother.** This shows generalized spike and wave complexes which are suggestive of generalized epileptic activity.

**Figure 2:**
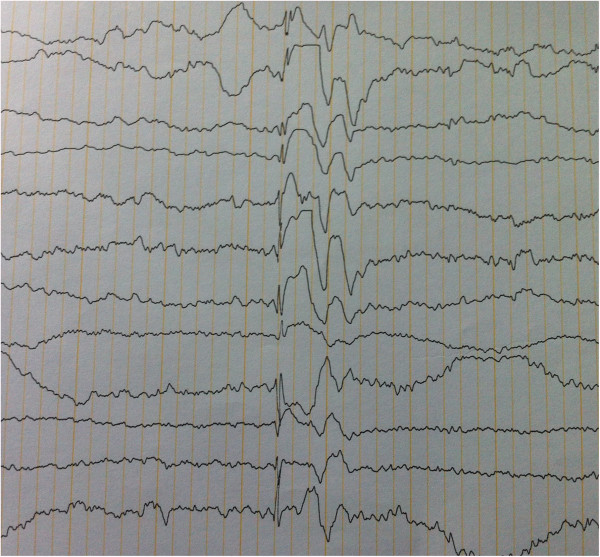
**EEG of the younger brother.** This is similar to his brothers EEG with generalized spike and wave complexes.

Investigation results were identical in both of them. Brian and spinal cord MRI did not reveal any structural lesions. Electroencephalogram (EEG) showed frequent, irregular and generalized spike and wave complexes which were suggestive of generalized epileptic activity (Figures 1 and 2). Paroxysmal activity was increased by intermittent photic stimulation and by hyperventilation. In all muscles examined, electromyogram (EMG) disclosed fasciculations, impaired recruitment with increased firing rate of the motor units and polyphasic, high-amplitude, long duration potentials. Motor and sensory conduction velocities were within the normal values. These features identified lower motor neuron degeneration. Muscle biopsy revealed a typical denervation pattern but no changes suggestive of a mitochondrial disorder. Serum creatine kinase level was mildly elevated. Genetic analysis was not done as it was not available in Sri Lanka for the relevant genes.

In order to obtain seizure control, sodium valproate and clonazepam were started. Rehabilitation with physiotherapy and occupational therapy was initiated.

## Discussion

Spinal muscular atrophies (SMAs) are a group of disorders characterized by loss of motor function and muscle atrophy due to anterior horn cell degeneration of the spinal cord and motor cells of lower cranial nerve nuclei. Four different types of SMA have been described depending on the age of onset and length of survival. However, overlap is present in some families, and most of these conditions are linked to the same chromosomal region, 5q11.2-q13.3 (SMN gene). Thus, they are considered allelic disorders with an earlier onset related to more frequent deletions in SMN gene [1–3]. Electrophysiological studies and muscle biopsy are of paramount importance since they allow confirming of motor neuron disease. Nowadays, genetic analysis allows definitive diagnosis. The two patients described in our case report has the late onset form, ie. Type IV SMA. As genetic analysis was not done in our patients, diagnosis of SMA was confirmed by typical clinical, EMG and histological findings which had been described earlier. Generalized spike and wave complexes on EEG (which reveals generalized epileptic activity) with myoclonus pointed towards myoclonic epilepsy. However there was no cognitive decline or ataxia even several years after the onset of myoclonus to suggest a syndrome of Progressive Myoclonic Epilepsy (PME).

Genetically different SMA variants, with additional or atypical clinical features, have been previously described in the literature and proved to be not linked to 5q [3]. In particular, clinical picture characterized by the co-existence of SMA and PME has been described rarely. Jancovic and Rivera first described a North American pedigree in which three members showed slight mental retardation, adult-onset myoclonic epilepsy, in association with SMA [4]. Striano and colleagues reported a case of childhood onset SMA, deafness, dysarthria, dysphagia and PME with absence seizures [1]. Lance and Evans reported a Finnish boy, who developed action myoclonus, generalized seizures and muscular atrophy during childhood with sensorineural hearing loss [5]. Marjanovic et al. described two brothers who showed proximal signs of SMA, action myoclonus and drug-resistant generalized epilepsy but not deafness [6]. Haliloglu et al. reported four patients from two families, showing a similar clinical picture to the two brothers of Marjanovic et al. and without evidence of SMN gene mutations [7]. Jie Zhou and colleagues reported three SMA-PME affected families with missense mutation or deletion of ASAH1 gene [8]. Dyment et al. reported an adolescent female who presented with atonic and absence seizures and myoclonic jerks and was later diagnosed as having myoclonic-absence seizures. Whole exome sequencing (WES) identified two rare, deleterious mutations in the ASAH1 gene. Functional studies in cultured fibroblasts showed that acid ceramidase was reduced in both overall amount and enzymatic activity. Ceramide level was doubled in the patient's fibroblasts as compared to control cells. The results of the WES and the functional studies prompted an electromyography (EMG) study that showed evidence of motor neuron disease despite only mild proximal muscle weakness. These findings expand the phenotypic spectrum of SMA-PME caused by mutations in ASAH1 [9]. Our patients presented with adult onset SMA associated with non progressive myoclonic epilepsy. There was no deafness or cognitive impairment as described in some of the previous cases. Most of the above authors reported the combination of SMA with PME in contrast to our case. They postulated that SMA with PME could be a rare form of PME where myoclonic epilepsy appeared later in the course of the disease.

From review of our case and the literature data, we suggest that the association of SMA with myoclonic epilepsy, similar to the combination of SMA with PME, may constitute a separate syndrome with a homogeneous clinical and EEG picture, not linked to SMN gene. It is likely that the maternal uncle of the two brothers had also suffered from a SMA like neuromuscular disorder. With the available data, it is difficult to presume whether he suffered from SMA or a type of SMA plus syndrome which was not diagnosed until his death. Taking in to consideration of an affected uncle and a healthy mother, X linked inheritance is a possibility even though there is no consanguinity. However, availability of facilities for genetic analysis would have been a great asset in defining the mode of inheritance and to decide whether this is a genetically separate syndrome or not. Another point is that it is still few years after diagnosing SMA and myoclonic epilepsy. Available literature regarding the cases of SMA with PME illustrate that myoclonic epilepsy starts within few years after the development of muscle weakness (with or without other features such as cognitive decline). However it is necessary to follow up these two patients on long term basis for the development of features of progressive myoclonic epilepsy.

In summary, this condition also seems to delineate a peculiar form of “SMA plus”. This concept has been recently developed since various SMA phenotypes with additional clinical features have been described: a predominantly distal form of SMA with severe respiratory distress (SMARD), SMA associated with arthrogryposis and congenital fractures, form with pontocerebellar hypoplasia [2, 3]. Unfortunately genetic analysis, acid ceramidase and ceramide levels were not done in our patients as the necessary facilities were not available. The collection of similar cases is needed to better define distinct phenotypes, in order to perform genetic studies as well as to identify new genes and molecular pathogenetic mechanisms involved in this condition.

## Conclusions

Even if the clinical picture of SMA is mainly dominated by the diffuse muscular atrophy, in some cases, patients may show associated, atypical clinical features (“SMA plus”). In particular, the association of SMA and progressive myoclonic epilepsy (PME) has been previously described. We present the identical clinical and electrophysiological data of two brothers with type IV SMA associated with a unique form of non progressive myoclonic epilepsy without any features to suggest a syndrome of PME. This association may constitute a separate syndrome with unique clinical and electroencephalographic findings even though further genetic studies are necessary.

## Consent

Written informed consent was obtained from the patients for publication of this case report and accompanying images. A copy of the written consent is available for review by the editor of this journal.
